# Serum microRNAs – potent biomarkers for radiation biodosimetry

**DOI:** 10.18632/oncotarget.24381

**Published:** 2018-02-02

**Authors:** Bartłomiej Tomasik, Wojciech Fendler, Dipanjan Chowdhury

**Affiliations:** Department of Biostatistics and Translational Medicine, Medical University of Lodz, Lodz, Poland; Department of Radiation Oncology, Harvard Medical School, Dana-Farber Cancer Institute, Boston, Massachusetts, USA

**Keywords:** microRNA, biomarker, biodosimetry, radiation

Exposure to high doses of radiation poses a significant threat to human life. The current methods of accurately predicting the biological effect of radiation are still dependent on the manifestation of symptoms or rely on technically complex and time-consuming tests such as the dicentric chromosome assay [1]. One of the primary limitations of this approach is that it cannot be easily implemented at the point of care, making efficient triage impossible. Using a commercial dosimeter, which needs to be worn at the time of the event, is similarly unfeasible in a population facing accidental radiation exposure. Therefore, there is an acute need for the identification of radiation-specific biomarkers that are capable of rapid predicting latent damage to various organs and systems immediately after radiation exposure.

MicroRNAs (miRNAs) are non-coding RNAs of 19-22 nucleotides that regulate diverse cellular processes, and serum circulating miRNAs have recently emerged as promising biomarkers for diagnosing various malignancies and predicting treatment responses [2]. Attempts have been made to use serum miRNAs as biodosimeters, however, the overlap and replicability of the published results were disappointingly low [3, 4]. The possible explanation for those discrepancies could be caused by differences between experimental models and applied methodologies of quantifying circulating miRNAs.

In 2015 our team identified a panel of serum miRNAs that were differentially expressed in response to total body irradiation (TBI) in C57BL/6J mice 24 hours after exposure [5]. The results indicate that the miRNA-based assay provided robust and repeatable results diagnostic of different doses of radiation, including a clinically-efficient separation of sublethal (6.5 Gy) and lethal doses (8 Gy) lethal doses. Additionally, they demonstrated that the serum profile of miRNAs associated with lethal irradiation reverted to normal range if amifostine - a radioprotective agent - was given before irradiation with the 8Gy dose. This suggested that the serum miRNA signature may serve not only as a biodosimeter but is indicative of the biological impact of irradiation linked to the irreversible destruction of bone marrow.

The results obtained in a mouse model are not however easy to translate into clinical practice. Given the impossibility to test the efficacy of our biomarkers in humans directly, we sought to develop an experimental model mimicking the high-dose irradiation scenario as accurately as possible. Thus, we decided to test our miRNA-based diagnostic tool for lethal irradiation in nonhuman primates (NHPs) [6]. We studied two large cohorts (a primary group of 48 and a validation group of 54 animals) of healthy NHPs (*Macaca mulatta*, Chinese substrain) subjected to total doses of radiation ranging from 5.8Gy (LD30/60) to 7.2Gy (LD70/60) during a drug trial on radiation mitigating agents. We identified seven miRNAs (miR-30a, miR-126, miR-133a, miR-133b, miR-150, miR-215 and miR-375) that were altered by irradiation in both mice and NHPs, proving that this pattern is evolutionarily conserved, most likely due to conserved transcription factor binding sites within promoter regions of genomic miRNA-coding sequences. Interestingly, bioinformatic analyses revealed seven transcription factors that are predicted to bind to the promoter sequences of all seven conserved radiation-responsive miRNAs in mice, macaques and humans. It was demonstrated that serum concentrations of the evolutionarily-conserved miRNAs miR-30a, miR-126, and miR-375 correlated with the efficacy of a radioprotective agent, γ-tocotrienol (GT3). However, the concentrations of remaining four conserved miRNAs showed a greater changed in expression change to radiation than that observed in controls. This suggests that the processes regulated by GT3 differ from those modulated by amifostine, however, this issue requires in-depth mechanistic studies. In addition, we developed a classification tool trimmed down to only three miRNAs (miR-133b, miR-215, and miR-375) which accurately distinguished NHPs exposed to radiation from those who were not. We also found striking evidence of miRNAs sex-dependent changes in response to radiation: miR-16-2 was associated with survival and its decreased expression was predictive of death both in males and females, however, the males showed half the pre- and post-exposure expression than females. Therefore, the design of an effective prognostic model required us to account for the sex of the animals, and by doing so we showed that we can reproducibly predict radiation-induced mortality in NHPs using a model consisting of miR-30a and miR-126. Further studies are required to determine whether radiation-induced serum miRNA signatures are tissue-specific or related predominantly to damage of the hematopoietic system.

Unresolved before miRNA-based tests or drugs may be put into clinical practice. The biological mechanism of secreting the miRNAs into the serum remains unknown, as are the regulatory mechanisms leading to differential expression of radiation-inducible miRNAs depending on sex and their complex interactions with radiation mitigating or radioprotective agents. Moreover, if the observed miRNAs are exosome-bound, they could actively participate in cell-to-cell transfer and play an active role in mitigating radiation-induced damage, rather than acting only as biomarkers. However, studies suggest that novel biodosimetric miRNA-based tools and even RNA-based drugs will be within our reach in the near future.

Furthermore, the utility of miRNAs in detecting and quantifying radiation exposure may be potentially translated to the field of radiotherapy. Our clinical paper showed that circulating serum miRNA profile changes in response to the delivered dose of radiation of radiation in patients treated for lung cancer [7], while another report confirmed that miRNAs could be used in personalized radiotherapy to achieve the best tumor control with minimal healthy tissue toxicity [8]. Such studies are of utmost importance because normal tissue toxicity prevents dose escalation in patients undergoing radiotherapy.

## References

[1] Waselenko JK (2004). Ann Intern Med.

[2] Hayes J (2014). Trends Mol Med.

[3] Cui W (2011). PLoS One.

[4] Menon N (2016). PLoS One.

[5] Acharya SS (2015). Sci Transl Med.

[6] Fendler W (2017). Sci Transl Med.

[7] Dinh TK (2016). Radiat Oncol.

[8] Korpela E (2015). Br J Cancer.

